# Short-term changes in ganglion cell complex in patients with COVID-19 treated with hydroxychloroquine

**DOI:** 10.5935/0004-2749.202100107

**Published:** 2021

**Authors:** Kemal Örnek, Emine Temel, Gökçen Özcan, Özkan Kocamış, Nazife Aşıkgarip

**Affiliations:** 1 Department of Ophthalmology, Kırşehir Ahi Evran University School of Medicine, Kırşehir, Turkey; 2 Department of Ophthalmology, Kırşehir Ahi Evran Training and Research Hospital, Kırşehir, Turkey

Dear Editor,

Severe acute respiratory syndrome coronavirus 2 (SARS-CoV-2) is a highly pathogenic human coronavirus that can cause serious life-threatening respiratory disease and multiorgan failure^(1)^. Despite the swift global spread of coronavirus disease 19 (COVID-19), no reliable clinical data are available to endorse any specific medical therapy. Organ-supportive therapy, including respiratory therapy, in acute pulmonary failure is recommended by physicians worldwide.

For supportive management, hydroxychloroquine sulfate (HCQ) is being widely used. Retinal toxicity is a well-known adverse effect of HCQ. It is generally irreversible and can worsen even after treatment is discontinued. Although no clear evidence of retinal toxicity has been found in patients with COVID-19, screening for HCQ-related retinal adverse effects has been a major concern throughout the pandemic.

We assessed short-term changes in the ganglion cell complex (GCC)-the retinal nerve fiber layer, the ganglion cell layer and the inner plexiform layer-by using spectral-domain optical coherence tomography (SD-OCT) in patients with COVID-19 who were treated with HCQ. We studied the eyes of 40 patients with moderate type COVID-19 (Group 1) and the eyes of 40 healthy participants (Group 2). All the patients had tested positive for SARS-CoV-2 by real-time reverse transcriptase polymerase chain reaction. The protocol was performed in adherence to the tenets of the Declaration of Helsinki and was approved by the institutional review board of Kırşehir Ahi Evran University.

**Figure 1:**
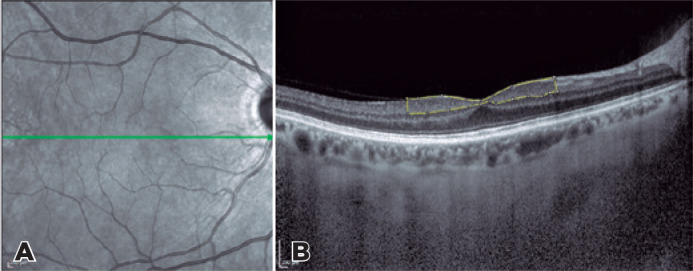
Spectral-domain optical coherence tomography image representing GCC measurement. (A) Scanning of the macular area (green line). (B) The macular volume of the ganglion cell complex including 3 retinal layers (yellow outline). The ganglion cell complex was defined as the area from the vitreoretinal interface to the inferior border of the inner plexiform layer and measured with the “analyze” tool in ImageJ software.

For the measurement, an area 3000 µm wide with a 1500-µm margin nasal to the fovea and a 1500-µm margin temporal to the fovea was selected. The GCC was defined as the area from the vitreoretinal interface to the inferior border of the inner plexiform layer and measured with the “analyze” tool in ImageJ software, version 1.50a (National Institutes of Health, Bethesda, MD, USA; Figure 1).

We found no significant differences in age (*p*=0.612) or gender (p=0.446). Slit-lamp examination yielded normal findings for all patients during the course of the disease and 6 months after recovery.

All patients received oral HCQ, 200mg twice daily for 5 days. GCC thickness was measured at baseline (0.227 ± 0.23 µm) and the end of the treatment (0.227 ± 0.22 µm). We found no significant difference in GCC thickness between the patients with COVID-19 and the controls (0.236 ± 0.22 µm) at baseline (p=0.143). Among the patients, GCC thickness did not change significantly from the first week to 6 months after recovery (0.233 ± 0.25 µm) (p=0.246). We also did not find a significant difference in GCC thickness between the patients and the controls 6 months after treatment (p=0.156).

HCQ, an antimalarial drug, is used in the treatment of autoimmune diseases such as systemic lupus erythematosus and rheumatoid arthritis. Retinal toxicity is the major and potentially most serious irreversible side effect of the treatment, occurring in approximately 3% of patients who take HCQ^(2)^. The risk of HCQ-related retinal toxicity is dependent on daily dose and duration of use. At recommended doses, the risk of toxicity for up to 5 years is less than 1%. If the daily dose is 4 to 5mg/kg, the risk for retinal toxicity is low during the first years of treatment. In our study, over a 5-day period, patients with COVID-19 received a high dose of HCQ, ranging from 200 to 400mg daily, according to different protocols. The safety of high doses of HCQ in a very short time period remains uncertain.

The mechanism of retinal toxicity is still unclear. In previous animal studies, the first histopathological changes were seen in the retinal ganglion cells^(3)^. One study of SD-OCT demonstrated thinning of the peripapillary layer of retinal nerve fibers and retinal ganglion cell axons in patients who had retinal toxicity^(4)^. Hallberg et al. reported that the effects on phospholipid metabolism after long-term chloroquine treatment of mice selectively involved retinal ganglion cells^(5)^.

The results of our study showed that HCQ use does not seem to produce a toxic effect on GCC during the acute phase of COVID-19 infection or 6 months after recovery. We believe that for patients with COVID-19 who were treated with HCQ, a digital medical record should be used to screen for toxicity in different organs over the long term. To understand the possible retinal effects of HCQ more precisely, longitudinal studies are needed.
